# Posttraumatic Stress Disorder and Autobiographical Memories in Everyday Life

**DOI:** 10.1177/2167702616688878

**Published:** 2017-03-13

**Authors:** Sabine Schönfeld, Anke Ehlers

**Affiliations:** 1 King’s College London; 2 University of Dresden; 3 Department of Experimental Psychology, University of Oxford; 4 Oxford NIHR Cognitive Health Clinical Research Facility

**Keywords:** posttraumatic stress disorder, autobiographical memory, overgeneral memory bias, trauma memories, intrusions, cognition

## Abstract

Evidence from self-reports and laboratory studies suggests that recall of nontrauma autobiographical memories may be disturbed in posttraumatic stress disorder (PTSD), but investigations in everyday life are sparse. This study investigated unintentional nontrauma and trauma memories in trauma survivors with and without PTSD (*N* = 52), who kept an autobiographical memory diary for a week. We investigated whether unintentional *nontrauma* memories show an overgeneral memory bias and further memory abnormalities in people with PTSD, and whether unintentional *trauma* memories show distinct features. Compared to the no-PTSD group, the PTSD group recorded fewer nontrauma memories, which were more overgeneral, more often from before the trauma or related to the trauma, were perceived as distant, and led to greater dwelling. Trauma memories were more vivid, recurrent, and present and led to greater suppression and dwelling. Within the PTSD group, the same features distinguished trauma and nontrauma memories. Results are discussed regarding theories of autobiographical memory and PTSD.

People with posttraumatic stress disorder (PTSD) suffer from unintentional intrusive and distressing memories of the traumatic event. These unwanted memories have attracted much attention in the theorizing and research on PTSD. Much less is known about spontaneous memories for other, nontrauma, autobiographical events in PTSD. There is some evidence that recall of nontrauma autobiographical memories may be disturbed in PTSD. Case reports suggest that some trauma survivors may develop retrograde psychogenic amnesia for some life periods (Markowitsch, 2002). More commonly, patients with PTSD describe that they have completely changed as a person since the traumatic event and have difficulty remembering what they used to be like (e.g., Ehlers, Maercker, & Boos, 2000). They often report that spontaneous remembering is mostly restricted to memories of the traumatic event (e.g., Ehlers, Hackmann, & Michael, 2004). Laboratory studies found an overgeneral memory (OGM) bias in PTSD similar to that observed in depression (for reviews, see Moore & Zoellner, 2007; Williams et al., 2007), that is, a bias in intentional nontrauma memories. So far, it remains unclear whether there are systematic differences in spontaneous everyday nontrauma memories in traumatized people with and without PTSD. This introduction briefly describes the characteristics of intrusive, unintentional *trauma* memories and subsequently focuses on theories and research on *nontrauma* memories in PTSD.

## Characteristics of trauma memories in PTSD

Intrusive, distressing, and unintentional memories of the traumatic event are a hallmark symptom of PTSD (American Psychiatric Association, 1994, 2013), and most theories of PTSD focus on explaining their development and maintenance (e.g., Brewin, 2014; Brewin, Dalgleish, & Joseph, 1996; Ehlers & Clark, 2000; Horowitz, 1986). These theories convergently address clinical observations that intrusive trauma memories seem to appear unexpectedly, lack a time perspective, contain mostly sensory aspects of a situation, and are associated with intense emotional reactions and subsequent avoidance.

Trauma memories in PTSD have mainly been investigated with retrospective questionnaires and interview assessments (e.g., Hackmann, Ehlers, Speckens, & Clark, 2004; Michael, Ehlers, Halligan, & Clark, 2005; Reynolds & Brewin, 1998, 1999; Speckens, Ehlers, Hackmann, Ruths, & Clark, 2007), which restricts the ecological validity of the findings. Even though the literature on meta-memory for naturally occurring events suggests that people’s retrospective judgments appear to be valid for salient and important events (Shlechter, Herrmann, & Toglia, 1990; but see Takarangi, Strange, & Lindsay, 2014, for conflicting findings), it would be desirable to obtain information on memories in people with PTSD as soon as possible after they occurred. A few studies have provoked intrusive memories by either presenting trauma-related pictures (Michael et al., 2005) or asking participants to give accounts of their trauma and to then subsequently identify flashback-like memories experienced during the narrative (Brewin, Huntley, & Whalley, 2012; Hellawell & Brewin, 2004; Whalley et al., 2013). These studies consistently reported that intrusive trauma memories in PTSD are particularly vivid and predominantly consist of sensory experiences, accompanied by high distress (e.g., Hackmann et al., 2004; Hellawell & Brewin, 2004). A finding of theoretical interest was that intrusive memories of trauma in PTSD appear to lack time perspective, that is, by being experienced as rather present as in the past (“nowness”; e.g., Hackmann et al., 2004; Hellawell & Brewin, 2004; Michael et al., 2005; Speckens et al., 2007). “Nowness” is seen as a central feature of memories of the worst moments during a trauma in cognitive models of PTSD (Brewin et al., 1996; Brewin, Gregory, Lipton, & Burgess, 2010; Ehlers & Clark, 2000) and is thought to result from a predominance of perceptual processing during trauma (Brewin, 2014; Ehlers & Clark, 2000; see also Kvavilashvili, 2014). The resulting memory is thought to be highly accessible by matching cues, poorly elaborated, disjointed from other autobiographical information, and retrieved with a lack of autonoetic awareness. There is some evidence that intentional retrieval of trauma memories is more disorganized in people with PTSD than those without PTSD (e.g., Halligan, Michael, Clark, & Ehlers, 2003; Jelinek, Randjbar, Seifert, Kellner, & Moritz, 2009). Berntsen and Rubin (2006) suggested that trauma memories are encoded as particular meaningful landmark events that become central to people’s life story. However, the question of whether in PTSD trauma memories are stored and remembered in a distinct way remains a debate (see Kvavilashvili, 2014, for a discussion).

Few studies to date have recorded trauma memories in people with PTSD promptly after they occurred. In two daily diary studies of intrusive memories of negative life events in student populations (Berntsen, 2001; Rubin, Boals, & Berntsen, 2008), students with PTSD-like symptoms rated their trauma memories compared to nontrauma memories as more vivid, as producing a worse mood and more physical reactions, and as more important (Berntsen, 2001). Rubin et al. (2008) reported that PTSD symptoms were associated with an enhanced availability of memories for distressing events for both voluntary and involuntary retrieval (also Berntsen & Rubin, 2014). Based on these results these authors (Berntsen & Rubin, 2014; Rubin et al., 2008) argued that intrusive remembering in PTSD might simply be explained by increased availability of the trauma memory due to general trait emotional reactivity to memories as a predisposing factor in vulnerable people. These authors predict differences between trauma and nontrauma memories to be continuous and trauma memories to be intense in both individuals with and without PTSD symptoms. In accord with this, McKinnon et al. (2014) found that enhanced memory for the trauma is not associated with symptoms of PTSD. However, these studies remain preliminary, as either they studied negative events that would not be considered traumatic (American Psychiatric Association, 1994, 2013) or results did not generalize when repeating subanalyses with traumatic events (Berntsen & Rubin, 2014), and PTSD symptoms were mild and were assessed by questionnaire only. Kleim, Graham, Bryant, and Ehlers (2013) used electronic diaries to compare unintentional trauma memories in people with and without PTSD. They found that for the PTSD group intrusive trauma memories appeared to happen in the here and now, and lead to stronger negative emotions such as fear or shame. Further data from daily recordings of clearly defined trauma memories in reliably diagnosed individuals are needed to validate the differences found in questionnaire and interview studies between trauma survivors with and without PTSD. Second, studies are lacking that directly compare involuntary trauma and nontrauma memories in traumatized persons with PTSD.

## Overgeneral memory bias in PTSD: Restricted to the laboratory or everyday problem?

Most studies of nontrauma autobiographical memories in PTSD have investigated OGM bias. This is usually assessed by applying the autobiographical memory test (AMT; Williams & Broadbent, 1986), where OGM shows as a reduction in the proportion of specific memories retrieved intentionally after cue presentation. Trauma survivors with PTSD or acute stress disorder (ASD) showed less specific intentional autobiographical memory retrieval than those without PTSD or ASD in the AMT (Harvey, Bryant, & Dang, 1998; Kleim & Ehlers, 2008; McNally, Lasko, Macklin, & Pitman, 1995; Nixon, Ball, Sterk, Best, & Beatty, 2013; Schönfeld & Ehlers, 2006; Schönfeld, Ehlers, Böllinghaus, & Rief, 2007; for reviews, see Moore & Zoellner, 2007; Ono, Devilly, & Shum, 2015). OGM in PTSD is found across different cultures (Graham, Herlihy, & Brewin, 2014; Humphries & Jobson, 2012; Jobson, Moradi, Rahimi-Movaghar, Conway, & Dalgleish, 2014), and OGM assessed soon after trauma predicted subsequent PTSD and major depression at 6 months over and above initial diagnoses and symptom severity (Bryant, Sutherland, & Guthrie, 2007; Kleim & Ehlers, 2008; but also see Hitchcock, Nixon, & Weber, 2014).

It is as yet unclear whether OGM in PTSD applies to spontaneous, unintentional memory retrieval in everyday life. The literature on autobiographical memory allows for different predictions. Unintentional memory retrieval is hypothesized to be a bottom-up process. This means that matching triggers may directly activate specific event representations (Conway & Pleydell-Pearce, 2000). The natural environment is rich in retrieval cues and direct retrieval may facilitate specific memories by bypassing retrieval on higher, abstract levels. In line with this suggestion, Berntsen (1996) found in a diary study of healthy volunteers that spontaneously occurring autobiographical memories in everyday life were more specific than those retrieved intentionally in the laboratory (also Schlagman & Kvavilashvili, 2008; but see Rubin et al., 2008, for conflicting findings). Watson, Berntsen, Kuyken, and Watkins (2013) compared the specificity of voluntary and involuntary autobiographical memories and potential interactions with depressive symptoms. Involuntary memories were more specific than voluntary memories. Nonremitted depressed participants showed reduced memory specificity for voluntary, but not for involuntary memories.

Those findings suggest that unintentional retrieval of autobiographical memories in PTSD may be unaffected by OGM bias, However, results of a study by Schönfeld and Ehlers (2006) cast doubts on this possibility. The authors used a pictorial version of the AMT, building on AMT research suggesting that imageability of cues and pictorial cues are associated with greater specificity (Belcher & Kangas, 2013; Williams, Healy, & Ellis, 1999), and found that OGM in PTSD generalized to pictorial cues.

There are theoretical and empirical reasons to suggest that OGM may apply to spontaneously occurring autobiographical memories in PTSD. The CaR-FA-X model (Williams et al., 2007) proposes that reduced executive functioning (X), rumination (R), capture (Ca), and functional avoidance (FA) are the central mechanisms explaining OGM in emotional disorders. The intentional search for specific memories in autobiographical memory retrieval is hypothesized to be a hierarchical, top-down process (Conway & Pleydell-Pearce, 2000; Williams & Dritschel, 1988) that, if aborted early, will result in an abstract general memory. Williams et al. (2006) suggested that rumination and abstract negative self-schemas may interfere with the retrieval of specific memories as people then get “captured” in abstract cognitions, such in as a “mnemonic interlock.” Similarly, the model suggests that retrieving general rather than specific memories reduces the emotional impact of remembering negative events, so that OGM may be functioning as an avoidance process in the face of adversity such as early trauma (i.e., Brennen et al., 2010; Crane et al., 2014; Ogle et al., 2013; Raes, Hermans, De Decker, Eelen, & Williams, 2003).

If OGM is linked to avoidance, several forms of cognitive avoidance may reduce the likelihood with which people with PTSD remember specific autobiographical events in their everyday life. Suppression of trauma memories, rumination about the trauma, and persistent dissociation have repeatedly been found to predict the persistence of PTSD (Ehring, Ehlers, & Glucksman, 2008; Murray, Ehlers, & Mayou, 2002) and are related to OGM in PTSD (Kleim & Ehlers, 2008; Schönfeld & Ehlers, 2006; Wessel et al., 2014). Schönfeld et al. (2007) found that an experimentally induced intention to suppress trauma memories increased OGM in nontrauma memories. They suggested that this may represent a “misguided suppression effect” in that a rebound in trauma memories leads to further (unsuccessful) suppression attempts, which then (successfully but dysfunctionally) generalize to other memories via a vicious cycle. An observation made by Hulbert, Henson, and Anderson (2016) supports this view. They found that retrieval of other than the to-be-suppressed material can be disturbed, potentially via disrupted hippocampal activity. Dalgleish, Rolfe, Golden, Dunn, and Barnard (2008) found in a differentiating experiment that associations between OGM and PTSD are most likely explained by emotion regulation tendencies (also see Bunnell & Greenhoot, 2012; Wessel et al., 2014). It is interesting and contrary that Raymaekers, Smeets, Peters, and Merckelbach (2010) found that OGM did not differ in abused persons when comparing those with recovered versus continuous memories of their abuse; however, their sample had very low PTSD levels, so that the proposed mechanisms might not have applied.

## Nontrauma memories: Further potential autobiographical memory alterations in PTSD

Studies investigating memory problems in PTSD yielded mixed results; most reliably replicated is a deficit in verbal memory (see Scott et al., 2015, for a review). There are very limited empirical data on autobiographical memory. McKinnon et al. (2014) found that for both trauma and nontrauma memories, people with PTSD retrieved more nonepisodic details of events than those without PTSD, but there were no differences in terms of richness or accuracy of nontrauma memories (see also Brown et al., 2014). Jelinek et al. (2009) found that nontrauma memory accuracy was related to trauma exposure rather than to PTSD symptoms. Blix and Brennen (2011) reported that trauma experience had no effect on the temporal distribution of autobiographical memories. Mixed results were found for valence effects and mood bias in PTSD for laboratory-induced memories (see Amir, Leiner, & Bomyea, 2010; but Moradi, Taghavi, Neshat-Doost, Yule, & Dalgleish, 2000), and it is unclear whether they apply to spontaneous autobiographical memories.

## Nontrauma memories and perceptions of the self

Traumatic experiences and subsequent symptoms may have profound effects on people’s sense of identity. Often the trauma is seen as a reference point in the person’s life (Berntsen & Rubin, 2006), and patients with PTSD have been described to be “frozen at the time” (Herman, 1992), “stuck” in the past (Holman & Silver, 1998), or “fixated to the time of the trauma” (McNally et al., 1995). It is thus possible that they have a bias in retrieving nontrauma memories that are nevertheless connected to the trauma. Furthermore, people with PTSD describe themselves as being permanently changed for the worse or feeling alienated from aspects of their life (Ehlers et al., 2000). Such changes in self-perception are most likely interwoven with altered autobiographical remembering. Correlational analyses suggested that OGM in PTSD is associated with perceived permanent change and dissociation (Kleim & Ehlers, 2008; Schönfeld & Ehlers, 2006). One may therefore expect that people with PTSD perceive spontaneous nontrauma memories as if they relate to a different self. Coping strategies such as persistent dissociation, thought suppression, and rumination may prevent individuals with PTSD from engaging with their everyday environment. This might contribute to the impression that their life is unreal (Halligan et al., 2003) and to a diminished encoding of new memories as well as a reduced spontaneous retrieval of stored autobiographical events.

## Aims of the study

The present study compared characteristics of naturally occurring autobiographical memories that traumatized people with and without PTSD experience in their everyday lives. The study had two aims:

1. To investigate whether traumatized people with PTSD show deficits in the retrieval of nontrauma memories compared to traumatized people without PTSD. We expected that people with PTSD would show a paucity of these memories, an OGM bias, and would experience these memories as distant from the self.
2. To investigate the characteristics of trauma memories in everyday life in people with PTSD. We expected that traumatized people with PTSD would experience more frequent trauma memories of greater vividness, nowness, and recurrent nature than those without PTSD. We also expected that people with PTSD would describe their trauma memories to a greater extent as landmark events in their biography than those without PTSD, and that they would try harder to suppress them and ruminate more about them. We expected that within the PTSD group, trauma memories would differ from nontrauma memories on the same dimensions.

## Method

### Participants

All participants had experienced a traumatic event that met Stressor Criterion A of the fourth edition of the *Diagnostic and Statistical Manual of Mental Disorders* (*DSM-IV*; American Psychiatric Association, 1994), that is, an event that involved actual threat of death or serious injury or physical integrity to self or others *and* the individual responded with intense fear or helplessness. Participants were recruited through local victim support organizations and emergency departments of local hospitals, via newspaper advertisements, and through an outpatient specialist service for psychological treatment of anxiety disorders.

A total of 75 traumatized adults were assessed for the study. Exclusion criteria were head injury, psychosis, and drug abuse or dependence. People were also excluded if they had experienced more than one trauma within the past five years to avoid ambiguity in the temporal relationship between their memories in regard to the trauma. In all, 21 people were excluded for the following reasons: drug abuse or dependence (*n* = 9), subthreshold PTSD (*n* = 6), psychosis (*n* = 2), multiple trauma (*n* = 2), failure to understand the diary instruction (*n* = 1), and inability to fill in the diary because of severe depression (*n* = 1). Two people decided not to participate in the study as they found it too effortful to keep the diary. Thus, the final sample comprised 52 participants. The PTSD group comprised 26 participants who met the diagnostic criteria for PTSD as assessed with the Structured Clinical Interview for *DSM-IV* (SCID; First, Spitzer, Gibbon, & Williams, 1995) and showed at least moderate PTSD symptom severity as indicated by a score of 17 or more on the Posttraumatic Diagnostic Scale (PDS; Foa, Cashman, Jaycox, & Perry, 1997). Two participants with PTSD had comorbid panic disorder, 3 agoraphobia, 1 social phobia, 3 specific phobia, 1 obsessive-compulsive disorder, 1 generalized anxiety disorder, 3 major depression, and 1 somatization disorder and hypochondriasis. Overall, 12 participants in the PTSD group had at least one comorbid Axis I disorder (46.2%).

The no-PTSD group comprised 26 participants who had experienced a traumatic event according to the *DSM-IV* but did not fulfill the diagnostic criteria for PTSD and did not score higher than 13 on the PDS. Two participants in the control group had one comorbid Axis I disorder (7.7%), 1 social phobia, and 1 somatization disorder. Table 1 presents the sample characteristics. The groups did not differ in age, sex, ethnicity, education, or trauma characteristics. As to be expected, the PTSD group reported greater severity of symptoms of PTSD, depression, and anxiety, had more comorbid diagnoses, and had lower verbal intelligence as measured by the Mill Hill Vocabulary Scale (Raven, Court, & Raven, 1994).

**Table 1.** Sample Characteristics

Variable	PTSD^a^	No-PTSD^a^	Statistic for group comparison
Age in years, *M* (*SD*)	41.5 (11.7)	35.2 (12.1)	*F*(1, 50) = 3.64, *ns*
Sex, *n* (%)			χ^2^(1, *n* = 52) = 1.26, *ns*
Female	13 (50.0)	17 (65.4)	
Ethnicity, *n* (%)			χ^2^(2, *n* = 52) = 3.45, *ns*
Black	7 (26.9)	2 (7.7)	*FI* (*n* = 52) = 4.91, *ns*
Caucasian	16 (61.5)	21 (80.8)	
Other	3 (11.5)	3 (11.5)	
Marital status, *n* (%)			χ^2^(3, *n* = 52) = 7.50, *ns*
Single	7 (26.9)	12 (48.0)	
In relationship	4 (15.4)	3 (12.0)	
Married	9 (34.6)	10 (40.0)	
Divorced/separated	6 (23.1)	0 (0.0)	
Missing data		1	
Education, *n* (%)			χ^2^(3, *n* = 52) = 6.04, *ns*
None	1 (3.8)	1 (3.8)	
Exams at age 16 (GCSE)	9 (34.6)	2 (7.7)	
Exams at age 18 (A level or equivalent)	4 (15.4)	4 (15.4)	
College or university	12 (46.2)	19 (73.1)	
Trauma, *n* (%)			χ^2^(3, *n* = 52) = 1.37, *ns*
Accidents	9 (34.6)	11 (42.3)	
Interpersonal violence	11 (42.3)	8 (30.8)	
Unexpected death of relative	3 (11.5)	2 (7.7)	
Other	3 (11.5)	5 (19.2)	
Injuries, *n* (%)			χ^2^(2, *n* = 52) = 2.70, *ns*
No injuries	7 (26.9)	12 (46.2)	
Minor injuries	11 (42.3)	10 (38.5)	
Major injuries	8 (30.8)	4 (15.4)	
Time elapsed since trauma, *n* (%)			χ^2^(3, *n* = 52) = 3.26, *ns*
< 6 months	4 (15.4)	1 (3.8)	*FI* (*n* = 52) = .396, *ns*
< 1 year	8 (30.8)	7 (26.9)	
< 2 years	7 (26.9)	6 (23.1)	
< 5 years	7 (26.9)	12 (46.2)	
PTSD symptom severity (PDS), *M* (*SD*)	30.08 (7.67)	4.19 (4.09)	*F*(1, 50) = 230.69***
Depression (BDI), *M* (*SD*)	20.35 (9.04)	4.04 (3.91)	*F*(1, 50) = 71.23***
Anxiety (BAI), *M* (*SD*)	23.19 (12.22)	5.27 (4.33)	*F*(1, 50) = 49.68***
Verbal Intelligence (MHV), *M* (*SD*)	19.00 (5.53)	23.42 (5.39)	*F*(1, 50) = 8.53**

*Note.* BAI = Beck Anxiety Inventory; BDI = Beck Depression Inventory; *FI* = Fisher’s exact test; MHV = Mill Hill Vocabulary Scale; *ns* = not significant; PDS = Posttraumatic Stress Diagnostic Scale.

a *n* = 26.

** *p* < .01. ***p < .001.

### Self-report measures

#### Posttraumatic Diagnostic Scale (PDS)

The PDS (Foa et al., 1997) is a standardized and validated self-report measure of PTSD symptom severity that has been widely used with clinical and nonclinical samples of traumatized individuals. The PDS asks participants to rate how much they have been bothered in the past month by each of the PTSD symptoms specified in *DSM-IV* ranging from 0 (*never*) to 3 (*5 times per week or more/very severe/nearly always*). Internal consistency (α) in the present sample was .95.

#### Beck Depression Inventory (BDI) and Beck Anxiety Inventories (BAI)

The BDI (Beck & Steer, 1987) and the BAI (Beck, Epstein, Brown, & Steer, 1988) assessed levels of depression and anxiety. Both instruments are of established reliability and validity. Internal consistencies (α) in the present sample were .92 (BDI) and .94 (BAI).

#### Mill Hill Vocabulary Scale (MHV)

The MHV (Raven et al., 1994) is a standard measure of verbal intelligence and asks participants to detect the correct synonym in a group of words. We administered Set A of the multiple-choice version of the senior form.

Table 1 shows the group descriptives for the demographic and clinical measures

#### Autobiographical memory diary

Participants described their autobiographical memories in a diary form for one week. Participants were asked to record all memories that appeared during this time and to give their ratings right after the memory occurred. The diary consisted of separate paper sheets (“memory sheets”), and each sheet was used to describe a single memory. A pilot study with 8 healthy volunteers and 4 PTSD patients tested the acceptability and wording of the diary form and determined the number of questions that could be asked about each memory. The final diary form assessed the following information about each memory: Participants noted the date and time of occurrence of each memory and gave a brief description of its content. They then answered questions about five groups of memory characteristics.

##### Relationship between memories and trauma

Participants indicated (a) whether the event had occurred before or after the traumatic event, or was the traumatic event, and (b) whether the content of the memory was related to the traumatic event.

##### Valence

Participants rated both the valence of the event and of the corresponding memory, on a scale from −5 (*very unpleasant*) to +5 (*very pleasant*).

##### Memory features

Participants reported (a) whether memory was intentional, on a scale from 0 (*not at all*) to 10 (*very much*), (b) how recurrent the memory was, on a scale from 0 (*had never before*) to 10 (*have had very often*), (c) how vivid the memory was, on a scale from 0 (*not at all*) to 10 (*very much*), and (d) how temporally remote the memory appeared, on a scale from 0 (*as if happening here and now*) to 10 (*long ago*).

##### Relationship of memory and the self

Participants rated (a) the extent to which the memory felt the event had happened to the same person they felt to be now, on a scale from 0 (*self different*) to 10 (*the same person*), (b) how likely the participant felt that this memory would occur as the first memory when thinking of that life period (landmark event), on a scale from 0 (*another one likely to come first*) to 10 (*this one likely to come first*), and (c) whether the memory was related to an important life theme, on a scale from 0 (*from not at all important theme*) to 10 (*very important*).

##### Reactions to memories

Participants reported (a) the extent to which they tried to push the memory out of their mind, on a scale from 0 (*push out*) to 10 (*hold in mind*), (b) how much time they spent thinking about the event after the memory occurred (not at all, a few seconds, about a minute, several minutes, 10 to 20 minutes, 20 minutes to an hour, and more than an hour), and (c) how many (if) connected memories they experienced (number to be written down). If a memory was followed by a “new” memory, a separate sheet was to be used and filled in.

The experimenter gave participants a few examples of autobiographical memories and gave detailed instructions on how to fill in the diary. Each item was explained and illustrated with an example. Participants were encouraged not to censor their memories, and told that when describing the content of a memory, they could use a description that revealed its meaning only to them. Participants also received the instructions in written form.

#### Trauma memory estimation

Before keeping the diary, participants estimated what percentage of their everyday memories they expected to be trauma memories.

### Procedure

The study was approved by the local ethics committee. Each participant gave written informed consent. Participants were then instructed in the usage of the memory sheet either over the telephone or in person, depending on their preference. They were given or sent the written instructions and memory sheets and rated the percentage of trauma memories expected for the diary week. After a week of recording, the participant attended a research session. The session started with an AMT (the results of which are presented in Schönfeld & Ehlers, 2006). The experimenter then systematically interviewed the participant about each memory entry in the diary, for each sheet respectively. This clarified whether the description was of an actual memory, helped determine the memory’s specificity, and clarified other open ratings or further questions. The session then continued with the MHV, symptom questionnaires, trauma interview, and the SCID. The participants were debriefed about the purpose of the study and received on average £35 as reimbursement for travel expenses and their time.

### Data analysis

Only diary entries that were clearly recognizable as memories were included in the analysis; therefore, thoughts and descriptions of current states, future worries, or prospective memories such as “I have to remember to take my medication” were excluded. As not all participants reported trauma memories in the diary, nontrauma and trauma memories were analyzed separately. Most memories were rated as unintentional. We first conducted separate analyses for *all memories* and *unintentional memories* only (operationalized as a rating less than 6 on the intentional scale). The results were the same except that group differences were more pronounced when the analysis was restricted to unintentional memories. For parsimony, we present only the results of the more conservative analysis using all memories.

Nontrauma memories were coded for specificity following Williams and Broadbent (1986). A memory was defined as specific if it was about “an event lasting a day or less, which occurred at a certain place and time even if the subject could not remember when,” as extended if it was about “an event lasting longer than a day,” and as general if the memory “reflected repeated activities” or if they were general memories about people or places. A random sample of 5% of the answers (*n* = 35) was rated by an independent second rater, who was blind to the diagnosis. Interrater reliability (κ) was .94.

Groups of related variables were analyzed with MANOVA, followed by univariate tests if the overall test was significant. Other variables were analyzed with ANOVAs. For variables correlating with MHV, we also tested whether the group difference remained significant if MHV was statistically controlled by ANCOVA. Frequencies of memories were log transformed and trauma-relatedness of memories square root transformed to normalize the distribution. To avoid distortions resulting from single memories for variables representing percentages of nontrauma memories (OGM, relationship to trauma), we included in the analysis of these variables only participants who had more than one nontrauma memory (*n* = 23 with PTSD, *n* = 26 without PTSD). The GLM procedure in SPSS 23 was used for data analyses. Significance levels were set at *p* < .05.

## Results

### Number of memories

Before starting the diary, the PTSD group predicted that a greater proportion of their everyday autobiographical memories would be trauma memories than the no-PTSD group, *M* = 52.7% (*SD* = 26.5%) versus *M* = 7.7% (*SD* = 11.7%), *F*(1, 49) = 62.36, *p* < .001, partial η^2^ = .560. The group difference remained significant when MHV was controlled for, *p* < .001. The predicted proportion of trauma memories correlated with the actual proportions recorded in the diary, *r* = .57, *p* < .001.

In the diary, the PTSD group reported a mean of 11.0 memories (*SD* = 7.6), and the no-PTSD group a mean of 21.4 memories (*SD* = 18.6), *F*(1, 50) = 8.031, *p* = .007, partial η^2^ = .138. All participants recorded at least one nontrauma memory, and 21 of the PTSD group and 16 of the no-PTSD group recorded at least one trauma memory in the diary. An ANOVA with the between-subject factor group (PTSD vs. no-PTSD) and within-subject factor memory type (trauma vs. nontrauma) showed a significant interaction, *F*(1, 50) = 44.35, *p* < .001, partial η^2^ = .470. Post hoc tests showed that the PTSD group reported fewer nontrauma memories, *F*(1, 50) = 18.06, *p* < .001, partial η^2^ = .265, but more trauma memories than the no-PTSD group, *F*(1, 50) = 11.07, *p* = .002), partial η^2^ = .181. MHV scores did not correlate with any of the frequency variables, all *r*s –.10 < *r* < .10.

### Characteristics of nontrauma memories

#### Overgeneral memory

The percentage of nontrauma memories that were classified as specific, general, or extended is shown in Table 2. The MANOVA showed a significant group difference, *F*(2, 46) = 3.96, *p* < .001, partial η^2^ = .147. The PTSD group reported fewer specific, *F*(1, 47) = 7.91, *p* = .007, partial η^2^ = .144, and more general memories in the diary than the no-PTSD group, *F*(1, 47) = 7.70, *p* = .008, partial η^2^ = .141. Group differences remained significant when MHV scores and age of memories were controlled by ANCOVA.

**Table 2.** Characteristics of Everyday Nontrauma Memories for the PTSD and No-PTSD Groups: Means and Standard Deviations

	PTSD^a^		No-PTSD^a^	
Variable	*M*	*SD*	*M*	*SD*
Total number of memories (*n*)	7.77	6.23	20.42	17.98
Memory specificity (%)				
Specific	50.31^b^	29.36	70.61	20.92
General	43.80^b^	32.20	23.24	18.64
Extended	5.89^b^	11.32	6.15	7.47
Relation to trauma (%)				
Before trauma	64.91^b^	28.39	45.39	24.17
After trauma	31.20^b^	26.36	51.36	23.14
Content related to trauma	26.21^b^	25.88	3.57	6.47
Valence (–5 to +5)				
Event	1.22	2.56	0.82	1.59
Memory	0.66	2.49	0.82	1.24
Memory features (0–10)				
Intentional	3.18	2.51	3.05	1.93
Recurrent	4.27	2.42	3.73	1.48
Vivid	7.55	1.96	6.15	1.42
Remote: Here & now (0) to long ago (10)	5.33	2.32	5.89	1.23
Memory and self (0–10)				
Self: Different (0) to same (10)	5.41	2.60	6.80	1.70
Landmark event	3.50	2.05	3.85	1.47
Important life theme	6.58	2.61	4.77	2.29
Reactions to memory (0–10)				
Suppression (0) to holding in mind (10)	6.56	3.37	5.48	1.11
Dwelling	3.85	1.16	3.17	0.79
No. of connected memories	1.45	1.27	0.84	0.88

a *n* = 26, if not otherwise specified. ^b^*n* = 23, as at least two memories were required for percentages.

#### Valence and relationship to trauma

Table 2 shows the characteristics of the nontrauma memories reported by the groups. There were no group differences in the valence of the events or the memories of them, *F*(2, 49) = 2.10, *p* = .133, partial η^2^ = .079. The MANOVA showed a significant group difference in the temporal relationship between the nontrauma memories and the trauma, *F*(3, 45) = 13.68, *p* < .001, partial η^2^ = .477. The PTSD group recalled fewer memories from after the trauma, *F*(1, 47) = 8.05, *p* = .007, partial η^2^ = .146, more memories from before the trauma, *F*(1, 47) = 6.76, *p* = .012, partial η^2^ = .126, and rated a higher proportion of their memories as trauma-related than in the no-PTSD group, *F*(1, 47) = 23.02, *p* < .001, partial η^2^ = .329.

#### Memory features

There was a trend for a group difference regarding the memory features, *F*(4, 47) = 2.31, *p* = .072, partial η^2^ = .164. The PTSD group reported that their nontrauma memories were more vivid than the no-PTSD group, *F*(1, 50) = 8.65, *p* = .005, partial η^2^ = .147.

#### Memory and self

Both groups differed regarding the self-related memory variables, *F*(3, 48) = 4.72, *p* = .006, partial η^2^ = .228. The PTSD group reported to feel less that they were the same self as in the memory than the no-PTSD group, *F*(1, 50) = 5.22, *p* = .027, partial η^2^ = .094, and rated their memories as related to more important life themes, *F*(1, 50) = 7.12, *p* = .010, partial η^2^ = .125.

#### Reactions to memories

Participants with PTSD differed from those without PTSD in their reactions toward nontrauma memories, *F*(3, 48) = 2.98, *p* < .042, partial η^2^ = .157. They spent more time thinking about the memory than those without PTSD, *F*(1, 50) = 5.92, *p* = .017, partial η^2^ = .108, and reported a higher number of connected memories, *F*(1, 50) = 4.08, *p* = .05, partial η^2^ = .075.

### Characteristics of trauma memories

Characteristics of the trauma memories reported in the diary are shown in Table 3.

**Table 3.** Characteristics of Everyday Trauma Memories for the PTSD and No-PTSD Groups: Means and Standard Deviations

	PTSD^a^		No-PTSD^b^	
Variable	*M*	*SD*	*M*	*SD*
Total number of memories (*n*)	3.23	3.71	0.96	1.15
Valence (–5 to +5)				
Event	−4.66	0.54	−3.55^c^	2.08
Memory	−4.39	1.15	−2.57^c^	1.92
Memory features (0–10)				
Intentional	1.35	1.88	2.13^c^	2.70
Recurrent	8.65	1.83	6.60	2.37
Vivid	8.37	2.24	5.57	2.92
Remote: Here & now (0) to long ago (10)	2.50	1.74	6.23	2.03
Memory and self (0–10)				
Self: Different (0) to same (10)	5.50	3.66	5.03	3.79
Landmark event	7.58	2.39	5.94^d^	2.74
Important life theme	6.95^e^	4.01	4.29^c^	4.12
Reactions to memory (0–10)				
Suppression (0) to holding in mind (10)	2.05	2.44	3.72^c^	2.11
Dwelling	4.40	1.08	3.08^c^	0.92
No. of connected memories	1.49^f^	1.18	0.93^d^	0.99

a *n* = 21; ^b^*n* = 16; ^c^*n* = 15; ^d^*n* = 14; ^e^*n* = 19; ^f^*n* = 20.

#### Valence

The MANOVA indicated a group difference in valence, *F*(2, 33) = 7.96, *p* = .002, partial η^2^ = .325. The PTSD group rated both their traumatic event, *F*(1, 34) = 5.55, *p* = .024, partial η^2^ = .149, and the trauma memories as more negative, *F*(1, 34) = 12.58, *p* = .001, partial η^2^ = .270, than the no-PTSD group.

#### Memory features

The groups differed in reported trauma memories features, *F*(4, 30) = 9.60, *p* = .001, partial η^2^ = .561. The PTSD group rated their trauma memories as more recurrent, *F*(1, 34) = 8.62, *p* = .006, partial η^2^ = .202, vivid, *F*(1, 34) = 10.60, *p* = .003, partial η^2^ = .238, and to a greater extent as appearing to happen in the present, *F*(1, 34) = 35.08, *p* < .001, partial η^2^ = .508, compared to the no-PTSD group. There were no group differences in whether or not the trauma memories were experienced as unintentional.

#### Trauma memories and self

There were no significant group differences, but there were trends for the PTSD group to report a greater landmark quality of their trauma memories, *F*(1, 33) = 3.55, *p* = .069, partial η^2^ = .097, and to report them as more related to important life themes, *F*(1, 32) = 3.61, *p* = .067, partial η^2^ = .101.

#### Responses to trauma memories

The groups differed in terms of their responses toward trauma memories, *F*(1, 30) = 7.68, *p* = .001, partial η^2^ = .434. The PTSD group reported that they made more efforts to suppress the memories, *F*(1, 34) = 4.63, *p* = .039, partial η^2^ = .120, and dwelled longer on them, *F*(1, 34) = 15.03, *p* < .001, partial η^2^ = .307.

### Trauma versus nontrauma memories in the PTSD group

The overall test revealed differences in pleasantness ratings between trauma and nontrauma memories, *F*(2, 19) = 57.70, *p* < .001, partial η^2^ = .859, event rating *F*(1, 20) = 90.46, *p* < .001, partial η^2^ = .819, memory rating, *F*(1, 20) = 121.45, *p* < .001, partial η^2^ = .859. Memories also differed in their features, *F*(4, 16) = 689.10, *p* < .001, partial η^2^ = .994. Trauma memories were more frequently recurring, *F*(1, 20) = 30.11, *p* < .001, partial η^2^ = .601, less intentional, *F*(1, 19) = 11.91, *p* = .003, partial η^2^ = .385, more vivid, *F*(1, 20) = 15.80, *p* = .001, partial η^2^ = .441, and more experienced as happening in the here and now, *F*(1, 20) = 32.25, *p* < .001, partial η^2^ = .617. Regarding self-relatedness, *F*(3, 16) = 102.07, *p* < .001, partial η^2^ = .950, they were rated as more a landmark event, *F*(1, 20) = 45.95, *p* < .001, partial η^2^ = .697; however, there were no differences in the other variables of this category. Participants’ reactions to trauma memories differed from those toward their nontrauma memories, *F*(3, 18) = 206.04, *p* < .001, partial η^2^ = .972. They thought longer about them, *F*(1, 20) = 8.19, *p* < .02, partial η^2^ = .291, suppressed them more, *F*(1, 20) = 18.26, *p* < .001, partial η^2^ = .482, and indicated having more connected memories, *F*(1, 20) = 7.33, *p* < .02, partial η^2^ = .268.

## Discussion

The present study was to our knowledge the first to compare everyday nontrauma autobiographical memories in traumatized people who met diagnostic criteria for PTSD compared to those without PTSD. It is also one of the few studies investigating features of everyday trauma memories in traumatized persons with and without PTSD with concurrent recordings. No other study to our knowledge using these methods allowed comparisons between both memory types within a group of persons with PTSD.

### Overgeneral memory retrieval in everyday life

In line with laboratory research showing an OGM bias in PTSD (see reviews by Moore & Zoellner, 2007; Ono et al., 2015), the nontrauma memories retrieved by the PTSD group were less specific and more general than those of the no-PTSD group. Thus, the present study extended the finding of OGM to everyday memories, including unintentional remembering.

OGM in PTSD thus appears to be independent of retrieval conditions. This is in line with our prior finding of OGM in PTSD when pictorial cues were used (Schönfeld & Ehlers, 2006). The pattern of findings suggests that access to specific memories is impaired in PTSD and that direct retrieval does not overrule this memory bias. This effect remained significant when controlling for differences in verbal intelligence and age of memories. To our knowledge, the only study to date investigating OGM in involuntary memories found OGM for voluntary but not for involuntary memories in patients with depression (Watson et al., 2013). Further studies are needed to clarify the difference in findings for OGM for involuntary memories between PTSD and depression.

The pattern of findings supported the rumination, capture, and avoidance hypotheses of OGM (Williams et al., 2007). Overgeneral retrieval as an abstract remembering style may mirror rumination as an abstract thinking style, both of which are related to PTSD. The PTSD group thought longer about their nontrauma memories and reported more connected memories than the no-PTSD group, suggesting that spontaneous memories triggered ruminative thought processes and further memories that were thematically linked (see Mace, 2014, for a theoretical discussion of memory chains). It should be noted, however, that the groups did not differ in the suppression of nontrauma memories. However, as Schönfeld et al. (2007) argued, it is possible that a repeated suppression of *trauma* memories might bring about suppression of other memories (“misguided suppression”; see also Hulbert et al., 2016).

An alternative explanation for OGM in everyday life could be that participants with PTSD are less aware of their environment and thus process cues that may trigger specific memories less efficiently than people without PTSD. Their general memories may have been mainly triggered by abstract internal cues, such as ruminative thoughts.

### Impoverished nontrauma memory retrieval in PTSD

The deficit in nontrauma memory recall in PTSD appeared to go beyond OGM in that traumatized people with PTSD recorded fewer nontrauma autobiographical memories in their everyday life than traumatized people without PTSD, and this was not explained by verbal memory deficits. Furthermore, a substantial proportion of these memories were related to the trauma. The PTSD group further showed a predominance of memories from the time before the trauma, and had fewer recent memories than the no-PTSD group (for different findings see Blix & Brennen, 2011). Thus, the pattern of results for the PTSD group deviates from the usual forgetting function (Ebbinghaus, 1885; Rubin, 1982), which in uncued autobiographical remembering in healthy individuals is also hypothesized to be invariant across different time scales (Maylor, Chater, & Brown, 2001; Moreton & Ward, 2010). A low retention of recent memories in PTSD may interfere with everyday functioning in that the ability to remember recent events may be necessary when adjusting to current task needs, such as problem solving (Anderson & Schooler, 1991; Berntsen, 1996). It might also interfere with contextualizing and “updating” trauma memories in that the person fails to process that nontraumatic, positive events after the trauma are also part of the biography, adding to a feeling of being permanently changed by the trauma (Kleim & Ehlers, 2008). Furthermore, in PTSD, despite being pretraumatic, those memories were nevertheless rated as related to the trauma, supporting the potential role of the trauma as a landmark event when thinking of other events retrospectively or the role of appraisals of significant change caused by the trauma. At this stage it is not possible, however, to differentiate whether the low recall of recent events in PTSD reflects a problem in retrieval or a problem in encoding.

### Other group differences in memories of nontraumatic events

People with PTSD reported that their nontrauma memories felt as if they happened to a different person and reported more frequently that the memory reflected an important life theme. Together with ruminative remembering style, the pattern of findings resembles those of a study by Teasdale and Green (2004), who found that ruminative and neurotic self-focus were linked to memories being experienced as “not at all at-one with things.” The authors see this as a processing style that is focused on discrepancies, contributing to feelings of hopelessness and depression. Another explanation could be that experiential alienation toward autobiographical memories is an epiphenomenon of dissociative symptoms in PTSD.

Our finding that people with PTSD experienced their nontrauma memories as more vivid than those without PTSD was unexpected, especially because these were more overgeneral. One explanation could be that memory overgenerality might reflect another dimension than lack of details per se. It is conceivable that event summaries, as in overgeneral remembering, do not necessarily lack visual details as details of several situations could still be merged into one “picture.” It is also possible that the vividness results from a perceptual processing style in people with PTSD that would enhance remembering of perceptual details of personal events. Perceptual processing has been discussed as a risk factor for PTSD (Ehlers & Clark, 2000). This suggestion is supported by McKinnon et al. (2014) who found enhanced details (but not richness) in both trauma and nontrauma memories in PTSD. Another explanation could be that the trauma-relatedness of these memories leads to an increase in arousal, potentially affecting vividness judgments of other memories as well. It is noticeable that we did not find any valence effect, suggesting that there is no general negativity bias for memories in PTSD.

### Characteristics of trauma memories in PTSD

As expected, participants with PTSD recorded more trauma memories than the No- PTSD group, and rated these as more frequently recurring. The difference was also present in their predictions of what proportion of trauma memories they expected to experience, taken prior to the diary week. The predicted ratio correlated with the actual ratio between trauma and nontrauma memories, suggesting that it might be a valid and possibly more informative measure of memory disturbance in PTSD that intrusion frequency (see also Michael et al., 2005).

Furthermore, trauma memories in PTSD were rated as more vivid and characterized by a sense of “nowness” than trauma memories in the no-PTSD group and nontrauma memories in the PTSD group. These results are in line with studies using retrospective self-reports of trauma memory characteristics as well as laboratory-induced intrusive trauma memories (Berntsen, Willert, & Rubin, 2003; Hackmann et al., 2004; Hellawell & Brewin, 2004) and concurrent monitoring (Kleim et al., 2013). The “nowness” of trauma memories might point to a lack of time perspective or “autonoetic awareness” in the PTSD group (Ehlers et al., 2004). “Nowness” of intrusive trauma memories has shown to be a better predictor of chronic PTSD than intrusion frequency (Michael et al., 2005), and this aspect of reexperiencing has been proposed as a defining characteristic of intrusions in the current conceptualization of PTSD in the 11th Revision of the International Classification of Diseases (Maercker et al., 2013). Ehlers and Clark (2000) proposed that “nowness” is a sign that those parts of the trauma that are reexperienced are poorly elaborated and thus susceptible to direct retrieval without context information. People may perceive an imminent threat to their lives when having intrusions of particularly distressing moments from the trauma as they do not simultaneously access relevant context information such as the fact that they have survived the trauma (see also Brewin et al., 2010, for similar suggestions linking these observations to neuroscience findings).

Contrary to our expectation, there was only a trend (partial η^2^ = .097 and .101, respectively) for the PTSD group to rate their trauma memories to a greater extent as landmark events and as related to an important theme than those without PTSD, partly supporting Berntsen’s (2001) findings. Low power and the assessment with only one item may have contributed to the nonsignificant finding. However, the PTSD group rated their trauma memories as landmark events to a greater extent than their nontrauma memories. Thus, there was some support for the hypothesis that traumatized people use the trauma as reference point when remembering other events from their lives (Berntsen & Rubin, 2006).

PTSD patients experienced their traumatic event and memories as more negative than did the no-PTSD group and as more negative than their nontrauma memories, even though both groups reported the same types of traumas and with no differences in trauma severity. Due to the cross-sectional nature of the study it is not possible to distinguish between PTSD as a consequence of a particularly negative event or the evaluation of the event as particularly negative possibly also as a consequence of subsequent symptoms.

It is interesting that the groups did not differ in terms of intentionality of both their trauma and nontrauma memories; however, the PTSD group experienced their trauma memories as less intentional than their nontrauma memories. Thus, trauma memories may be similarly involuntary for individuals after a trauma, yet be judged as pathological or predominant in particular by persons with PTSD, possibly contributing to negativity ratings. Cognitive models of PTSD propose that negative interpretation of symptoms is a maintaining factor for PTSD symptoms (i.e., Ehlers & Clark, 2000).

Participants with PTSD also thought longer about their trauma memories and tried to push them out of their mind to a greater extent than those without PTSD. These results are in line with previous results highlighting the role of rumination and thought suppression in maintaining PTSD (e.g., Ehlers, Mayou, & Bryant, 1998; Ehring et al., 2008; Kleim, Ehlers, & Glucksman, 2007). The PTSD group also reported retrieving more connected memories than the no-PTSD group, which could mirror a general ruminative style in PTSD as discussed earlier.

### Study limitations

The present study was a field study, leading to several methodological difficulties. First, it is not possible to determine whether participants recorded all memories they experienced. Some participants may have found it aversive to record unpleasant memories, and others may have failed to record fleeting memories of other more trivial events. Factors influencing monitoring compliance such as motivation or competing mental demands may have affected whether or not a particular memory was recorded. This may restrict the validity of the findings, although it is unlikely that the particular pattern of findings is explained by such biases. Furthermore, the proportion of intrusive trauma memories recorded in the diary corresponded well with the participants’ prior estimates. Should the PTSD group have avoided recording trauma memories, the present results reflect a conservative assessment of their frequency.

Second, assessing OGM in the natural environment deviates from the standard AMT test setting. There was no time limit and no prompting, or usage of standardized cues, and participants had not particularly been instructed to produce specific memories. Conservatively, our finding of OGM in PTSD can therefore only be interpreted as an everyday retrieval pattern rather than a lack of ability in retrieving specific memories.

Third, the present study used a cross-sectional, correlational design so that causal conclusions cannot be drawn. Prospective longitudinal studies could help elucidate the temporal relationship between altered everyday memory characteristics and PTSD.

Fourth, the study had a relatively small sample size so that unexpected findings that are not in line with the previous literature should be treated with caution. Further studies are needed to replicate our findings.

## Conclusions and Clinical Implications

Overall, this study of participants with clinically significant traumas showed several alterations of everyday autobiographical memories in people with PTSD. Participants with PTSD had problems accessing events from their lives other than the trauma, even when unintentional memories were considered, and their nontrauma memories were mainly related to their trauma. They experienced older, fewer, and more general nontrauma memories than traumatized people without PTSD. These results are of theoretical and clinical interest.

The results indicate that OGM bias is not restricted to intentional remembering, which is at odds with current theories suggesting that OGM does not apply to unintentional remembering (Williams et al., 2007). The study also showed for the first time that OGM bias in unintentional remembering is related to posttrauma psychopathology. Further research is needed to replicate the results and investigate their specificity to PTSD. Theories of autobiographical memory need to explain how the presence of symptoms of PTSD, but not trauma alone, is associated with profound changes in everyday memory including a paucity of nontrauma memories. Although theories of PTSD have focused mainly on characteristics of the development and maintenance of intrusive trauma memories, our results suggest that this approach may be too narrow to understand the influence of memory function on PTSD symptoms. The value of the characteristics of involuntary nontrauma memories observed in this study in predicting PTSD warrants further investigation. These may contribute to symptom development or maintenance by preventing trauma memory contextualization and integration, and may therefore be promising targets for intervention. The infrequent and overgeneral retrieval of nontrauma memories in PTSD may also contribute to the interference of the disorder with the patients’ lives. It contributes to a changed sense of self, which has been shown to maintain PTSD, and may also lead to depression and hopelessness (Kleim & Ehlers, 2008). The ability to retrieve memories of events from our past helps grounding ourselves in relation to time and place, which is a necessary condition when trying to act effectively in our environment and in respect to future-oriented goals (Pillemer, 2003). Further research should investigate whether OGM and paucity of involuntary nontrauma memories can be altered with treatment.

Trauma memories in PTSD showed distinct features (greater involuntariness, nowness, landmark quality, negativity, suppression, and dwelling), compared to the no-PTSD group, suggesting that there are features of trauma memories in PTSD that cannot solely be explained by having experienced trauma (see also Brewin, 2014). These results support existing theories and treatments of PTSD that focus on elaborating the moments in memory that are involuntarily reexperienced to reduce their nowness, and on changing unhelpful responses to memories such as suppression and dwelling (e.g., Brewin, 2014; Ehlers & Clark, 2000).

It appears plausible that the memory alterations in trauma and nontrauma memories interact with each other. These considerations suggest that interventions that counteract the retrieval difficulties and help PTSD patients remember past experiences that are unconnected to the trauma may be helpful. Moradi et al. (2014) recently reported positive effects of a memory specificity training in PTSD (“MEST”, Raes, Williams & Hermans, 2009). However, the results remain preliminary as this training has not yet been compared to a placebo condition and only been tested with rather small samples. It is heavily based on training the AMT-typical correct responses. Our findings suggest that an autobiographical memory training could be possibly even more effective if it addressed further aspects of autobiographical remembering than just overgenerality. A first additional target for intervention is to enhance access to nontrauma memories by providing retrieval cues. Some empirically validated treatments for PTSD include interventions that facilitate access to memories of prior, functional aspects of their lives (e.g., cognitive therapy for PTSD—Ehlers, Clark, Hackmann, McManus, & Fennell, 2005; narrative exposure therapy—Schauer, Neuner, & Elbert, 2011). The present findings suggest that access to more recent non-trauma-related memories should also be enhanced. If present, dysfunctional cognitions about autobiographical remembering in general can be addressed with cognitive therapy, for example appraisals that the self is permanently damaged or lost, that difficulties in memory retrieval indicate brain damage, or that allowing the traumatic memory might be dangerous (Ehlers & Clark, 2000). Following this, a further target is a reduction in suppression and dwelling tendencies, for example with mindfulness-based approaches or cognitive behavioral techniques.
